# Knowledge domain and hotspots analysis concerning applications of two-photon polymerization in biomedical field: A bibliometric and visualized study

**DOI:** 10.3389/fbioe.2022.1030377

**Published:** 2022-09-30

**Authors:** Hongxun Fu, Xian Jing, Jieqiong Lin, Liye Wang, Hancheng Jiang, Baojun Yu, Meiyan Sun

**Affiliations:** ^1^ Key Laboratory of Micro/Nano and Ultra-precision Manufacturing, School of Mechatronic Engineering, Changchun University of Technology, Changchun, Jilin, China; ^2^ College of Pharmacy, University of Houston, Houston, TX, United States; ^3^ College of Laboratory Medicine, Jilin Medical University, Changchun, Jilin, China

**Keywords:** two-photon, photopolymerization, bibliometrics, biomedical, citespace, bibliometrix, visualized

## Abstract

**Objective:** Two-photon polymerization (TPP) utilizes an optical nonlinear absorption process to initiate the polymerization of photopolymerizable materials. To date, it is the only technique capable of fabricating complex 3D microstructures with finely adjusted geometry on the cell and sub-cell scales. TPP shows a very promising potential in biomedical applications related to high-resolution features, including drug delivery, tissue engineering, microfluidic devices, and so forth. Therefore, it is of high significance to grasp the global scientific achievements in this field. An analysis of publications concerning the applications of TPP in the biomedical field was performed, and the knowledge domain, research hotspots, frontiers, and research directions in this topic were identified according to the research results.

**Methods:** The publications concerning TPP applications in biomedical field were retrieved from WoSCC between 2003 and 2022, Bibliometrics and visual analysis employing CiteSpace software and R-language package Bibliometrix were performed in this study.

**Results:** A total of 415 publications regarding the TPP applications in the biomedical field were retrieved from WoSCC, including 377 articles, and 38 review articles. The studies pertaining to the biomedical applications of TPP began back in 2003 and showed an upward trend constantly. Especially in the recent 5 years, studies of TPP in biomedical field have increased rapidly, with the number of publications from 2017 to 2021 accounting for 52.29% of the total. In terms of output, China was the leading country and Chinese Acad Sci, Tech Inst Phys and Chem was the leading institution. The United States showed the closest cooperation with other countries. ACS applied materials and interfaces was the most prolific journal (*n* = 13), followed by Biofabrication (*n* = 11) and Optics express (*n* = 10). The journals having the top cited papers were Biomaterials, Advanced materials, and Applied physic letters. The most productive author was Aleksandr Ovsianikov (27 articles). Meanwhile, researchers who had close cooperation with other researchers were also prolific authors. “cell behavior”, " (tissue engineering) scaffolds”, “biomaterials,” and “hydrogel” were the main co-occurrence keywords and “additional manufacturing”, “3D printing,” and “microstructures” were the recent burst keywords. The Keyword clusters, “stem cells,” and “mucosal delivery”, appeared recently. A paper reporting unprecedented high-resolution bull models fabricated by TPP was the most locally cited reference (cited 60 times). “Magnetic actuation” and “additive manufacturing” were recently co-cited reference clusters and an article concerning ultracompact compound lens systems manufactured by TPP was the latest burst reference.

**Conclusion:** The applications of TPP in biomedical field is an interdisciplinary research topic and the development of this field requires the active collaboration of researchers and experts from all relevant disciplines. Bringing up a better utilization of TPP as an additive manufacturing technology to better serve the biomedical development has always been the research focus in this field. Research on stem cells behaviors and mucosal delivery based on microstructures fabricated using TPP were becoming new hotspots. And it can be predicted that using TPP as a sourcing technique to fabricate biomedical-related structures and devices is a new research direction. In addition, the research of functional polymers, such as magnetic-driven polymers, was the frontier topic of TPP biomedical applications.

## Introduction

Two-photon polymerization (TPP) utilizes an optical nonlinear absorption process to initiate the polymerization of photopolymerizable materials. The light source used by TPP is usually near-infrared femtosecond laser. By focusing the femtosecond laser on the photoresist, a photoinitiator (PI) molecule in the photopolymer is excited by absorbing two photons at the same time (i.e., two-photon absorption), thereby inducing local radical polymerization in the focal volume (Jing et al., 2022). TPP is an additive manufacturing (AM) technology, which belongs to the category of optical three-dimensional printing (3DP) (Jonušauskas et al., 2018). However, due to the threshold and properties of TPP, compared to the common used optical 3DP techniques such as selective laser sintering (SLS), stereolithography (SLA), and digital light processing (DLP), the 3D structures fabricated by TPP has higher spatial resolution and can exceed the diffraction limit. Structures with a resolution of less than 100 nm have been repeatedly reported (Sakellari et al., 2012; Jing et al., 2022). Meanwhile, the wavelength of the laser used for TPP is transparent to the photopolymer processed, so the focus can move freely in the material, which makes this technique gets rid of the limitations of layer-by-layer processing of other 3DP technologies and realize truly three-dimensional arbitrary writing (Baldacchini et al., 2021). Therefore, compared with other high-resolution manufacturing technologies, such as traditional photolithography, dip-pen nanolithography, capillary force lithography, nanoimprint lithography, and others, features available in 3D structures fabricated using TPP would be comparable to what can be achieved in 2D. In addition, compared with traditional 3D manufacturing technologies applied in the biomedical field, including particulate leaching, solvent casting, freeze-drying, electrospinning etc., TPP not only has higher processing resolution but also can more precisely reproduce complex artificially designed 3D structures (Jing et al., 2022). TPP is currently the only technique capable of fabricating complex 3D microstructures with finely adjusted geometry on the cell and sub-cell scale. Because of its unique and superior characteristics, TPP has shown a very attractive promise in biomedical applications related to high-resolution features, including drug delivery, tissue engineering, microfluidic devices and so forth (Raimondi et al., 2012). Bibliometrics based on computer science has become an effective tool in scientific research, artificial intelligence (AI) assisted instruction and other relevant fields. It is a discipline to analyze the knowledge domain, hotspots, frontiers, and development trends of a particular research field (Gonzalez-Alcaide et al., 2017). Being an advanced approach for scientific analysis of publications (Chen, 2004; Chen and Song, 2019), bibliometrics focuses on the metrology of the features of the countries, institutions, journals, authors, keywords, and cited literature (Ellegaard and Wallin, 2015). A remarkable development in the field of bibliometrics is the application of information visualization analysis of publications, which enables people to have a concrete and clear perception of abstract knowledge by generating networks and maps of scientific research information in specific fields (Bederson and Ben 2003; Gonzalez-Alcaide et al., 2017). This said, information visualization based on bibliometrics provides an objective and scientific judgment on the connotation and extension of publications, helping people to quickly grasp the scope and theme, the history and current situation, the frontiers and trends in a relevant scientific research field (Chen, 2006; Chen and Song, 2019). Bibliometrics and visualized studies have been widely applied in many disciplines and fields and achieved a multitude of results (Ke et al., 2020; Liu et al., 2021; Dong et al., 2022; Xiong et al., 2022). However, the bibliometric and visualized study for the TPP technique is still lacking, especially for the applications of TPP in biomedical field.

In this study, we applied bibliometric analysis with the assist of the bibliometrics software CiteSpace and the R-language package Bibliometrix to analyze the worldwide publications concerning the applications of TPP in the biomedical field. We analyzed and identified the knowledge domain, research hotspots and frontiers in a relevant field, and shed light on the future directions based off our research outcomes.

## Materials and methods

### Data source and search strategy

We completed a targeted search of English literature in Web of Science Core Collection (WoSCC) in the field of TPP biomedical applications from 1 January 2003 to 1 August 2022. Web of Science Core Collection (WoSCC) is the preferred database for bibliometrics analysis. We found that there was merely a little literature published before 2003 in related research field. Following concepts were used to conduct the queries: [(TS= (“two photon” polymerization OR “two photon” lithography OR “two photon” crosslink*) AND (TS= (medic* OR biolog* OR biomedic* OR “tissue engineer*" OR “drug delivery")] and the English literature types were filtered as “article” or “review article”. The qualified literature was selected for further bibliometric analysis, and the record content was “full record and cited references”.

### Statistical analysis

In this study, CiteSpace (version 5.8. R3), and the Bibliometrix 4.2.1 *R* Package, and an online bibliometric analysis platform (https://bibliometric.com) were used to perform the bibliometrics analysis.

Bibliometrix is an R-tool for comprehensive science mapping analysis of bibliometrics (Aria and Cuccurullo, 2017). Using Bibliometrix, we conducted the analysis of overall information, journals analysis and analysis of institutions, and obtained trend topics and milestone literature concerning the publications of the field of related research.

CiteSpace is a visual analysis software of bibliometrics, which is able to analyze the knowledge field, research scope, hotspots, and frontiers of specific research fields (Chen, 2004; Chen and Leydesdorff, 2014). Using CiteSpace, we conducted the analysis of dual-map overlay, keywords analysis, references analysis, and cluster analysis for the articles concerning the research topic studied by this paper. It is remarkable that cluster analysis has two evaluation indexes, modularity Q and mean silhouette value. When the values of these two indexes are more than 0.3 and 0.5 respectively, the clustering results are clear and reliable.

In addition, the online bibliometric analysis platform (https://bibliometric.com) is applied to assist in analyzing the degree of cooperation between countries in the biomedical applications field of TPP.

It is necessary to note here that to more accurately analyze the knowledge domain, hotspots, and frontiers in the field studied in this article, the information from 377 articles was used in terms of dual-map overlay, references analysis, keywords analysis and co-authors analysis, and 415 papers (articles and reviews) were used in the analysis of other aspects.

## Results

### Overall information

**FIGURE 1:**
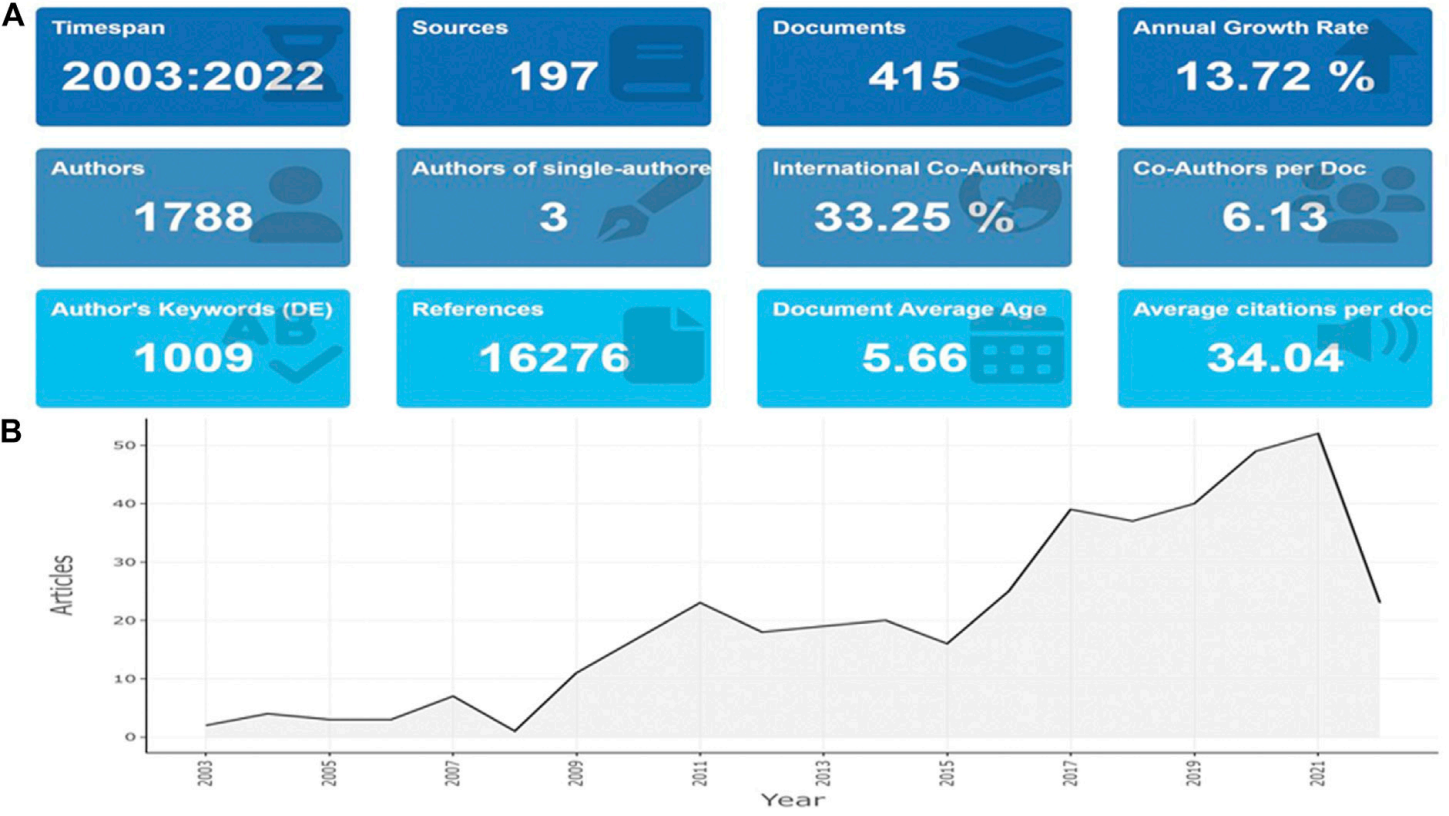
Main information **(A)** and annual scientific production **(B)** of publications concerning applications of TPP in biomedical field.

A total of 415 hits meeting the eligibility criteria were found and retrieved from WoSCC, including 377 articles, and 38 review articles. Figure 1A shows the main information of the screened publications. It indicated biomedical applications of TPP was a fairly new research area (document average age = 5.66). As shown in Figure 1B, the studies on biomedical applications of TPP began in 2003 and were associated with an upward trend on the whole. The annual increase rate of publications was 13.72%. Typically, in recent 5 years, studies of TPP in biomedical field had a great deal of increase by 52.29% from 2017 to 2021.

### Leading countries

**FIGURE 2:**
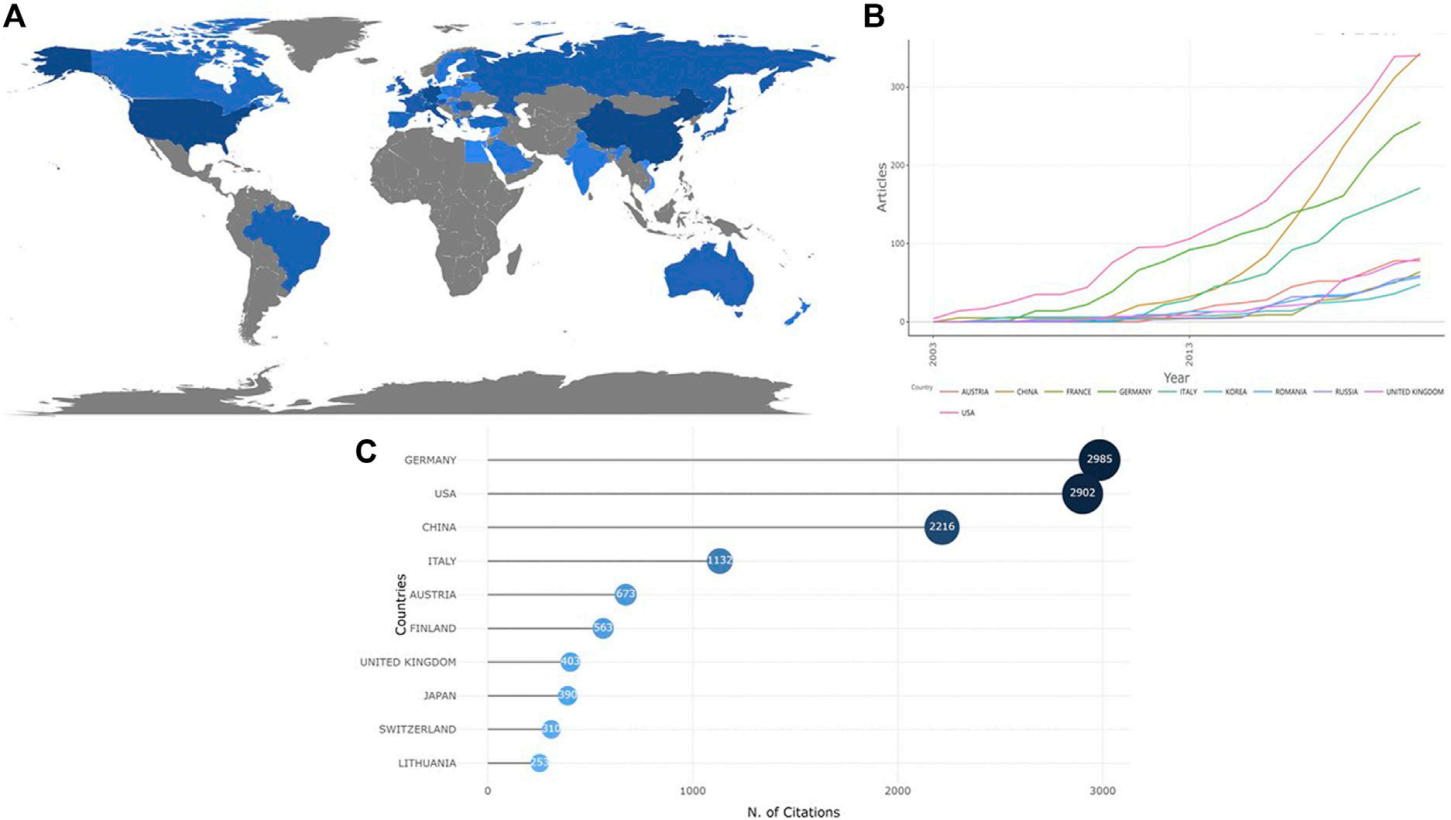
Contributions of different countries regarding the research of TPP applications in biomedical field. **(A)** Global country scientific production contributions; **(B)** Production of the top 10 countries with the highest productivity over time; **(C)** Number of publications in the top 10 countries with the highest productivity.

**FIGURE 3:**
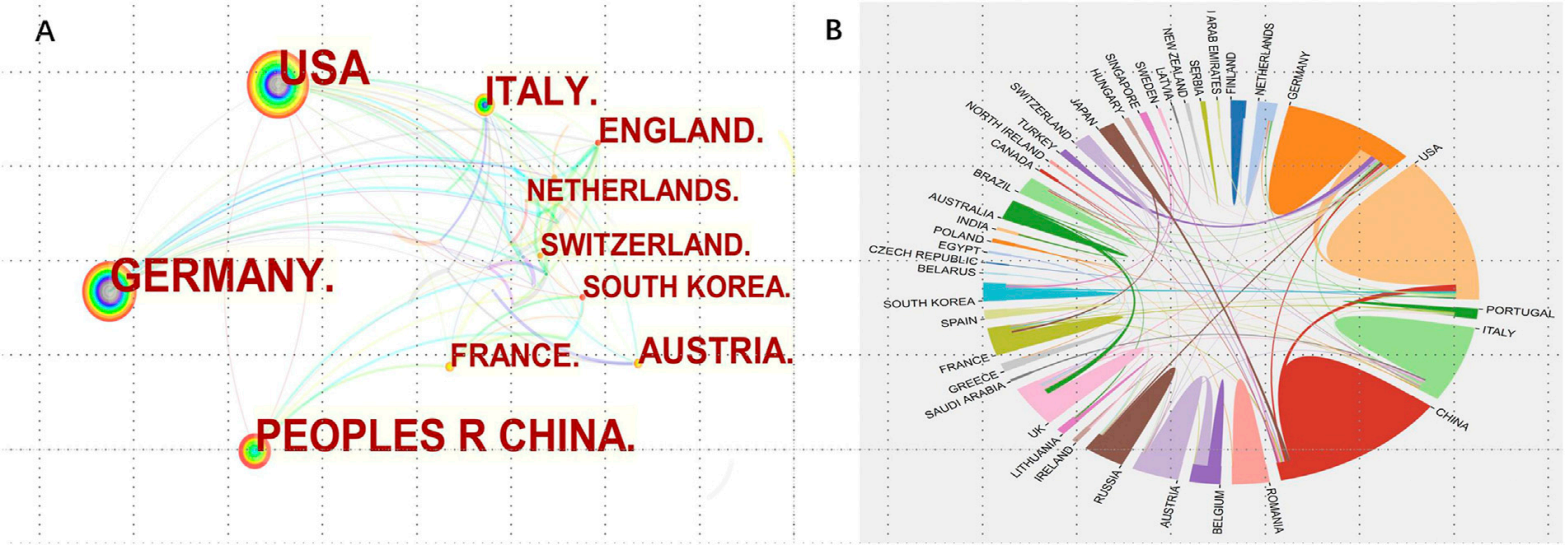
Cooperation of countries with regard to applications of TPP in biomedical field. **(A)** The network of cooperative relations between countries generated with CiteSpace; **(B)** Visualized map of cooperative relations between countries (established with https://bibliometric.com).

Since 2003, 39 countries had published papers on the applications of TPP in biomedical field. Global country scientific production is indicated in Figure 2A. As shown in Figure 2B and Table 1, the number of papers published by the top 10 countries with the highest productivity accounted for 76.2% of the total, of which China, the United States and Germany ranked the top three, having 352 (17.67%), 351 (17.62%), and 255 (12.80%) papers respectively. The most cited publications were from Germany (cited 2,985 times), followed by the United States (cited 2,902 times), and China (cited2216 times) (Figure 2C). Top 3 countries with the highest average number of citations were Turkey (164 times), Finland (112.6 times), and U Arab Emirates (76 times) (Table 2). As shown in Figure 3A,B, which visualize the international collaboration relations, the collaboration between different countries was relatively close. Among those, the United States showed the closest cooperation with other countries, followed by Germany and China. It should be pointed out here that to better display the international collaboration, Figure 3B was generated using the online bibliometric analysis platform https://bibliometric.com.

**TABLE 1:** Top 10 countries with the highest productivity of publications related to applications of TPP in biomedical field.

Rank	Country	Freq	Contribution (%)
1	China	352	17.67
2	United States	351	12.62
3	Germany	255	12.80
4	Italy	171	8.58
5	United Kingdom	81	4.07
6	Austria	78	3.92
7	France	64	3.21
8	Russia	59	2.96
9	Romania	57	2.86
10	South korea	50	2.51

**TABLE 2:** Top 10 countries with the highest average citations of published articles on TPP biomedical applications.

Rank	Country	TC	Average article citations
1	Turkey	164	164.00
2	Finland	563	112.60
3	U Arab Emirates	76	76.00
4	Poland	61	61.00
5	Switzerland	310	51.67
6	Lithuania	253	50.60
7	Germany	2,985	50.59
8	Singapore	198	49.50
9	Austria	673	44.87
10	Denmark	177	44.25

### Analysis of institutions and authors

**FIGURE 4:**
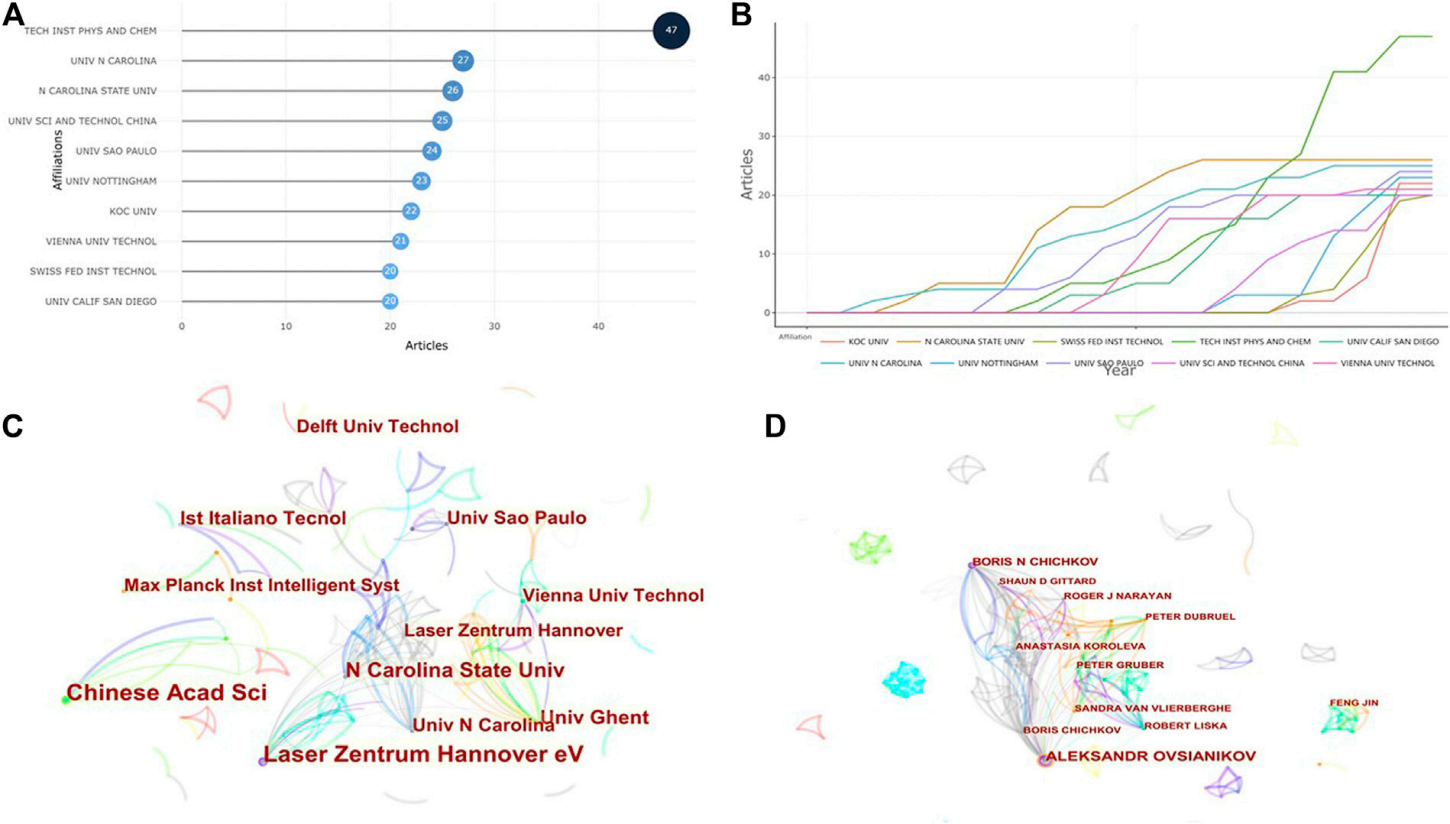
Visual analysis of institutions and authors concerning publications of TPP applications in biomedical field. **(A)** The top 10 institutions with the most published papers; **(B)** Production of the top 10 institutions with the highest productivity over time. **(C)** The network of cooperative relations between institutions. **(D)** The network of cooperative relations between authors.

Six hundred and twenty five institutions worldwide had participated in the research on TPP biomedical applications. The top 10 affiliations with the most published papers are shown in Figure 4A. Chinese Acad Sci, Tech Inst Phys and Chem (*n* = 47) was the leading institution in terms of output, followed by Univ N Carolina (*n* = 27) and N Carolina State Univ (*n* = 26). The research in this field did not start in Chinese Acad Sci, Tech Inst Phys and Chem until 2011, but the number of papers published by this institution increased rapidly, and the N Carolina State Univ carried out this research earlier and continued to this day (Figure 4B). The collaboration between various institutions was not very close, especially the cooperation across continents (Figure 4C). Comparatively, there was more cooperation between research institutions in European and American countries, and the institutions that published more papers also had more cooperation with other institutions. In addition, according to the co-authors’ network, the most productive author was Aleksandr Ovsianikov (27 articles), followed by Boris N Chichov (13 articles). As presented in Figure 4D, researchers who had close cooperation with other researchers were often prolific authors.

### Journals analysis

**FIGURE 5:**
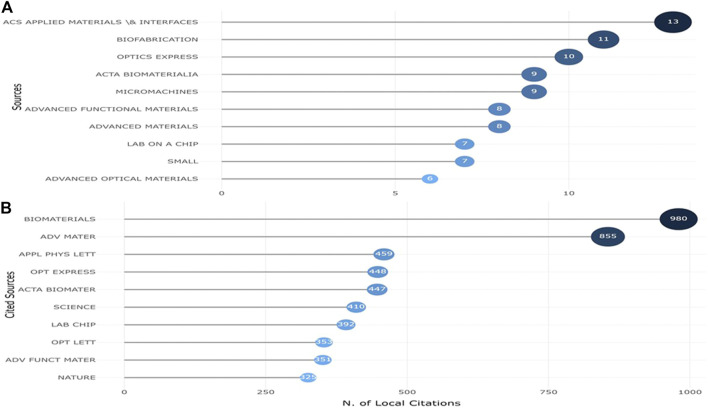
The top 10 most productive journals **(A)** and top 10 journals with the most cited publications **(B)** related to applications of TPP in biomedical field.

All papers were published in 197 journals. The top 10 journals with highest number of publications in our search are shown in Figure 5A, these journals published 21.20% out of total articles. ACS applied materials and interfaces was the most prolific journal (*n* = 13), followed by Biofabrication (*n* = 11) and Optics express (*n* = 10). Most local cited sources from reference lists are shown in Figure 5B, and the top 3 journals with the most cited papers were Biomaterials, Advanced materials and Applied physic letters.

**FIGURE 6:**
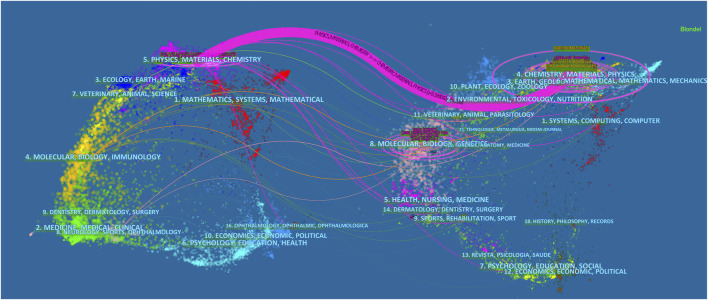
The dual-map overlay of journals related to applications of TPP in biomedical field.

Main citation paths are indicated by the dual-map that shows the topic distribution of journals. The left side of the dual-map overlay is the citing journals, the right side is the cited sources, and the color stripes in the middle indicate the citation paths (Chen, 2017). One main path and several minor paths had been displayed (Figure 6). The main path shows that the studies published in Physics/Materials/Chemistry journals were usually cited in the studies published in Chemistry/Materials/Physics journals. In addition, the studies, published in Molecular/Biology/Genetics journals, were usually cited in the studies, published in Molecular/Biology/Immunology and Medicine/Medical/Clinical journals.

### Keywords analysis

The research topics or special issues of papers are displayed through keywords, which are the core of academic papers (Qi-Qi et al., 2016). Therefore, keyword analysis can draw the research objects, contents, and hotspots of a certain research field.

**FIGURE 7:**
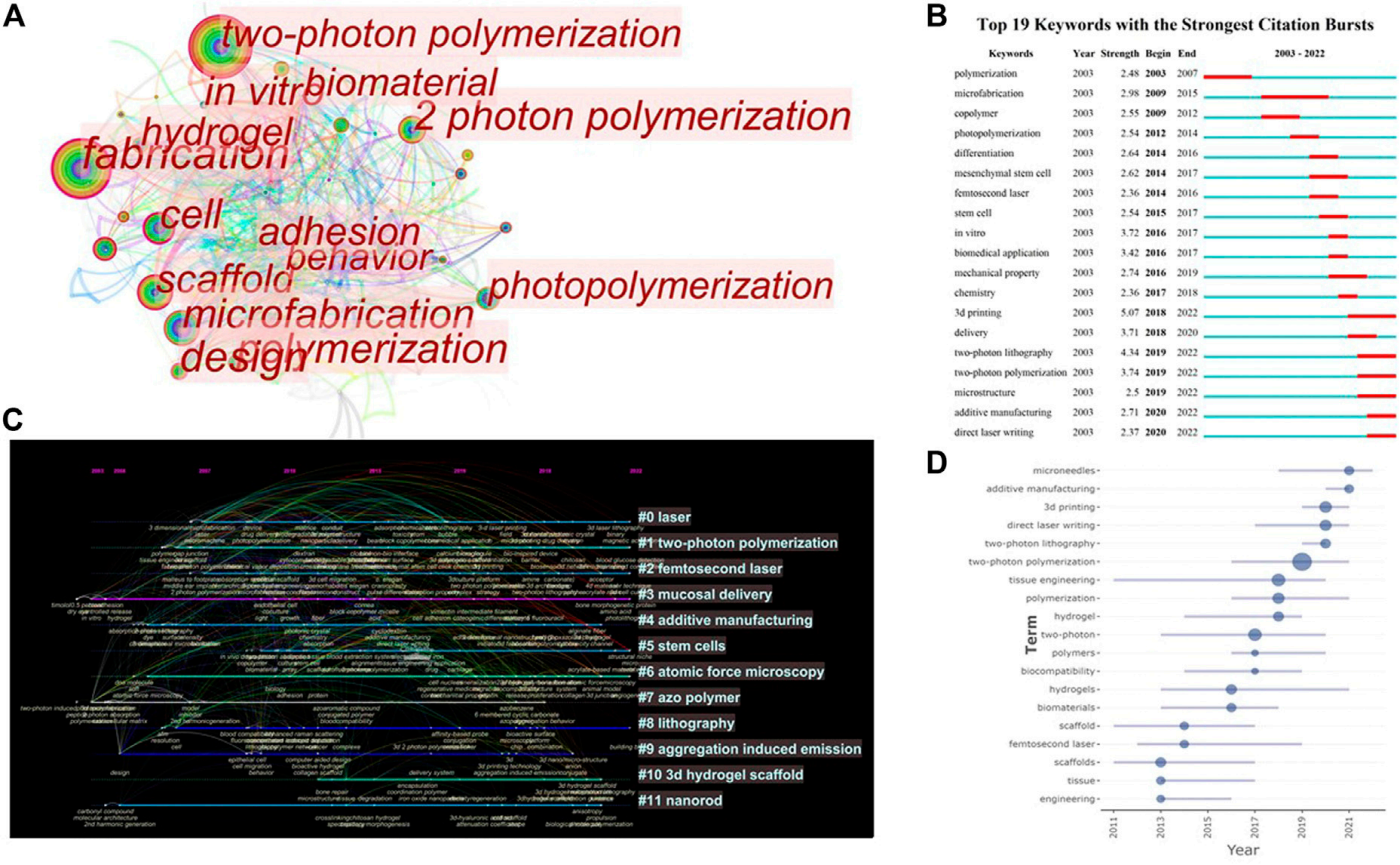
Visual analysis of keywords in publications on applications of TPP in biomedical field. **(A)** The keywords co-occurrence network; **(B)** Keywords burst analysis indicated by the map of “Top 19 Keywords with the Strongest Citation Bursts”; **(C)** The timeline of clustering for keywords; **(D)** Map of keywords trend topics.

Analyzing the literature data, we found that there were 415 keywords in 377 articles. The keywords co-occurrence network (Figure 7A) shows that TPP, as a “(micro) fabrication” technique, was applied to biomedical field, and its research contents were closely related to “cell behavior”, " (tissue engineering) scaffolds”, “biomaterials”, “hydrogel,” and so forth. Keyword bursts analysis (Figure 7B) shows that “copolymer”, “microfabrication”, “stem cells”, “*in vitro*” cells culture, and “(drug) delivery” had all been the hotspots of TPP applications in biomedical. In recent years, the research hotspot was still how to make better use of TPP as “additional manufacturing” and “3D printing” technology to fabricate “microstructures.”

From the modularity Q of a cluster was 0.5948 and the mean silhouette value was 0.8221, we can know that it was significant and reliable for CiteSpace to divide keywords into 12 clusters (Figure 7C). The timeline of clustering indicates that, as an optical “additive manufacturing” technology, the applications of TPP in biomedical field has always been the important content of research, whereas the research of fabrication for “stem cells” structural niches, and “mucosal delivery” were emerging hotspots in this field. In addition, we can conclude that, fabrication of “microneedles” using TPP was one of the important research hotspots recently by combining the analysis of keywords trend topics map generated *via* Bibliometrix (Figure 7D).

### References analysis

When literature A and literature B are cited simultaneously by literature C and other literature, A and B are called co-cited. By analyzing the cited literature and co-cited literature, we can provide intuitive tools for exploring the knowledge domain and frontiers of scientific research fields (Chen et al., 2010; Qi-Qi et al., 2016).

The top 10 most cited references are shown in Table 3. Most of the references locally cited frequently were groundbreaking articles. In 2001, Kawata et al. (2001) reported an unprecedented high-resolution bull model (10 μm long and 7 μm high) fabricated by TPP. This milestone breakthrough demonstrated the great potential of TPP for microfabrication and rapidly attracted the extensive attention of researchers. This paper was the most cited in the biomedical applications field of TPP (cited 60 times). Two pioneering papers describing the basic principle and process of TPP were also frequently cited (cited 57 times and 46 times) (Maruo et al., 1997; Cumpston et al., 1999). In addition, Tayalia et al. (2008) fabricated engineered polymer scaffolds using TPP to study 2D and 3D cell migration (cited 31times), and Ovsianikov et al. (2011c) demonstrated 3D tissue engineering scaffolds fabricated of Poly (ethylene glycol) diacrylate (PEGDA) by TPP(cited 31times). In addition to the fabrication of cell culture scaffolds, Doraiswamy et al. (2006) fabricated other microstructured medical devices using TPP (cited 30 times). It is noteworthy to mention that, in 2007, the first use of TPP to manufacture 3D porous scaffolds was demonstrated by the research team of Ovsianikov (cited 28 times) (Ovsianikov et al., 2007), which also developed a non-shrinking Zr-based hybrid organic-inorganic TPP processable material suited for biomedical applications (cited 29 times) (Ovsianikov et al., 2008).

**TABLE 3:** The top 10 most cited references cited by publications on applications of TPP in biomedical field.

Rank	Cited references	Citations
1	KAWATA S, 2001, NATURE, V412, P697, DOI 10.1038/35089130	60
2	MARUO S, 1997, OPT LETT, V22, P132, DOI 10.1364/OL.22.000132	57
3	CUMPSTON BH, 1999, NATURE, V398, P51, DOI 10.1038/17989	46
4	OVSIANIKOV A, 2011, ACTA BIOMATER, V7, P967, DOI 10.1016/J.ACTBIO. 2010.10.023	31
5	TAYALIA P, 2008, ADV MATER, V20, P4494, DOI 10.1002/ADMA.200801319	31
6	DORAISWAMY A, 2006, ACTA BIOMATER, V2, P267, DOI 10.1016/J.ACTBIO. 2006.01.004	30
7	SERBIN J, 2003, OPT LETT, V28, P301, DOI 10.1364/OL.28.000301	30
8	OVSIANIKOV A, 2008, ACS NANO, V2, P2257, DOI 10.1021/NN800451W	29
9	CLAEYSSENS F, 2009, LANGMUIR, V25, P3219, DOI 10.1021/LA803803M	28
10	OVSIANIKOV A, 2007, J TISSUE ENG REGEN M, V1, P443, DOI 10.1002/TERM.57	28
11	LEE KS, 2008, PROG POLYM SCI, V33, P631, DOI 10.1016/J.PROGPOLYMSCI. 2008.01.001	25
12	RAIMONDI MT, 2013, ACTA BIOMATER, V9, P4579, DOI 10.1016/J.ACTBIO. 2012.08.022	25
13	LAFRATTA CN, 2007, ANGEW CHEM INT EDIT, V46, P6238, DOI 10.1002/ANIE.200603995	24
14	OVSIANIKOV A, 2011, BIOMACROMOLECULES, V12, P851, DOI 10.1021/BM1015305	24
15	RAIMONDI MT, 2012, J APPL BIOMATER FUNC, V10, P56, DOI 10.5301/JABFM. 2012.9278	24
16	OVSIANIKOV A, 2012, EXPERT REV MED DEVIC, V9, P613, DOI 10.1586/ERD.12.48, 10.1586/ERD.12.48	22

**FIGURE 8:**
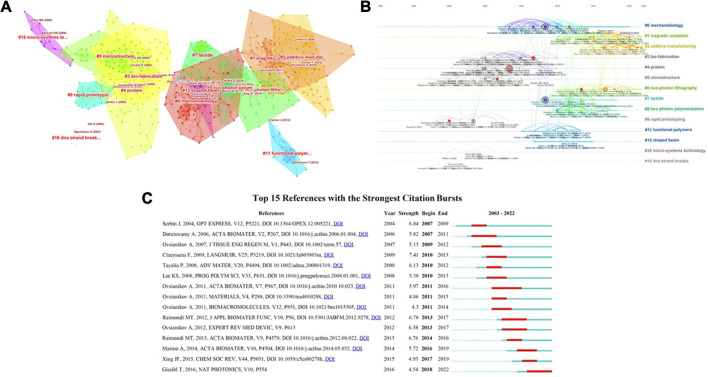
The analysis of references in publications on applications of TPP in biomedical field. **(A,B)** The visualized network and clustering timeline of the co-cited references; **(C)** Top 19 References with the Strongest Citation Bursts.

The visualized network and clustering timeline of the co-cited references were generated by CiteSpace (Figures 8A,B). A total of 19 clusters were constructed, which included “mechanobiology”, “magnetic actuation”, “additive manufacturing,” and “functional polymer”, among others. The modularity Q was 0.8459, and the mean silhouette value was 0.922. (Claeyssens et al., 2009), (Raimondi et al., 2012), and (Ovsianikov et al., 2011c), (Ovsianikov et al., 2012) were the top 3 papers co-cited most (Claeyssens et al., 2009). Reported for the first time the processing of biodegradable synthetic materials using TPP (Raimondi et al., 2012) and (Ovsianikov et al., 2012) are reviews concerning applications of TPP in biomedical field. Figure 8B shows “protein”, “functional polymer” and so forth were early fields, and recent frontiers were “magnetic actuation”, and “additive manufacturing” in biomedical applications of TPP. Moreover, we analyzed the references with citation bursts (Figure 8C), which can indicate the research hotspots in specific fields (Huang et al., 2019). In Figure 8C, the article of Serbin et al. (2004) has the highest burst strength. This paper demonstrated the 3D nanostructures fabricated using TPP, which fully showed the microfabrication ability of TPP, indicating the great potential of the TPP applications in biomedical field. In addition, the citation burst reference in recent years was a paper concerning ultracompact compound lens systems manufactured by TPP (Gissibl et al., 2016). The lens systems had shown unprecedented high-quality characteristics, which would have a profound impact on the biomedical field.

## Discussion

**FIGURE 9:**
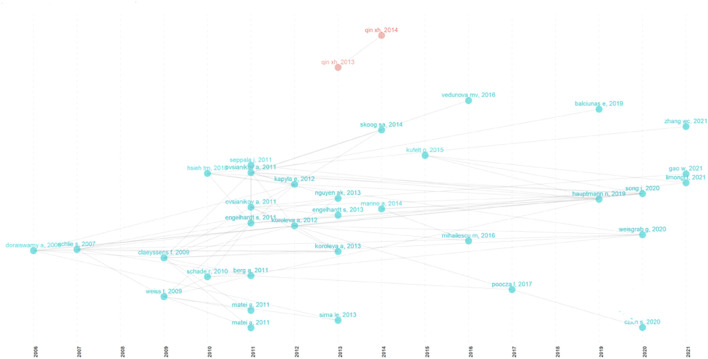
Landmark articles related to applications of TPP in biomedical field.

In this study, we found 415 articles that scoping on the applications of TPP in the biomedical field, with the earliest published back in 2003. We analyzed the knowledge domain, hotspots and emerging research trends on the screen publications and performed bibliometrics analysis to identify some landmark articles (Figure 9). As mentioned above, since Kawata et al. achieved a breakthrough in structural resolution using TPP in 2001, progress have been made in the applications of TPP in biomedical field. Especially after the first milestone literature demonstrating microstructured medical devices fabricated by TPP was published in 2006 (Doraiswamy et al., 2006), although there were fluctuations in individual years, the publications in this research field showed a stable upward trend. In 2011, landmark articles were published the most. In that year, both commercial photopolymers (ORMOCERs, a silicate-based organic-inorganic hybrid material) and naturally-derived materials (methacrylamide-modified gelatin) were utilized to fabricate 2D and 3D tissue engineering scaffolds using TPP, and multiple experimental results were achieved (Engelhardt et al., 2011; Matei et al., 2011; Ovsianikov et al., 2011a; Ovsianikov et al., 2011b). Subsequently, Kufelt et al. (2015) developed a water-soluble photopolymerizable chitosan hydrogel and fabricated cell culture scaffolds using TPP, further expanding the applications range of TPP biomaterials. The latest important research of TPP applications in biomedical field were the fabrication of 3D vessel analogs, and the formation of 3D micro-scaffolds that can be precisely adjusted for porosity to study modulation of the cell behaviors (Limongi et al., 2021; Zhang et al., 2021). It is worth noting here that, in addition to several articles describing the principle of TPP, there was a high degree of coincidence between the most local cited literature and landmark literature, indicating that the pioneering works possess great reference and guiding significance.

China was the most productive country, followed by the United States and Germany. The cooperation between these three countries and other countries was also the closest. Two of the top 5 productivity institutions come from China. Chinese Acad Sci, Tech Inst Phys and Chem (*n* = 47) was the leading affiliation in terms of output. The research team of Tech Inst Phys and Chem has long been committed to the development of water-soluble two-photon initiators for biomedical applications and the fabrication of hydrogel 3D microstructures, which are used to study cell behaviors (Yu et al., 2019; Zheng et al., 2019). The most cited country for publications was Germany. The research team of Hanover University in Germany is the first to use TPP to fabricate tissue engineering scaffolds (Schlie et al., 2007). The team also participated in other pioneering explorations such as the first use of TPP for the fabrication of 3D structures made of gelatin derivatives (Ovsianikov et al., 2011a) and the first printing of living cells to the 3D scaffolds fabricated by TPP (Schlie et al., 2007). Meanwhile, the authors who published more articles were generally from the countries and institutions that had been cited most or researchers who cooperated closely with these institutions. From the above qualitative and quantitative analysis, we can know that cooperation is very beneficial to output, whether between countries, institutions or authors. At the same time, we can also see from the above analysis that China, as the largest developing country, the United States, as the largest developed country, and Germany, as the output of many pioneering works in this field, had very close cooperation with other countries, which is easy to understand. However, in the institutions cooperation network, we can also see that the cooperation between European countries and European and American countries was closer, while China, as the most productive country in this research field, had relatively less cooperation with the institutions of developed countries in Europe and America. Therefore, in the future, China should strengthen cooperation with developed countries in Europe and the United States to accelerate the development of scientific research in this field.

Notably, the top 3 journals with the most articles scoped materials science, manufacturing science, and physics journals, indicating that the biomedical TPP applications is an interdisciplinary subject. In addition, two of the top 3 journals with the most cited papers were in materials science, implying that materials are crucial for the biomedical applications of TPP. The main citation path indicated by the dual-map further illustrates that this is an interdisciplinary research field, due to most of the cited sources and citing sources being both chemistry, materials, physics and biomedicine-related journals. For example, a recent paper discussed TPP for 3D biomedical scaffolds from the perspectives of physics, chemistry, material science, and biomedicine (Jing et al., 2022). This also shows that the development of TPP applied in biomedical field requires the full cooperation of experts from all relevant disciplines.

As pointed out by the analysis of keywords co-occurrence and co-cited references, TPP has been applied in biomedical field as an additive manufacturing technology since it was developed. Producing different structures to meet the needs of the biomedical field by using different biomaterials was the main knowledge domain concerning applications of TTP in biomedical field. From the above analysis, we can know that the production of different structures by using different biomaterials to meet the needs of the biomedical field is the main knowledge scope about the application of TTP in the biomedical field. This will also be the main research content of this field in the future. Drug delivery microneedles, microswimmers for biomedical applications, elements for microfluidic chip, and tissue engineering scaffolds have been fabricated by TPP (Amato et al., 2012; Maciulaitis et al., 2015; Wang et al., 2018; Cordeiro et al., 2020). With the development of TPP applications in biomedical field, some new hotspots and trends were gradually emerging. As shown by references and keywords bursts, and timeline of keywords clustering, how to make better utilization of TPP as an additive manufacturing technology to better serve the biomedical field has always been the research focus in this field. In addition, using TPP to fabricate microstructures for stem cells culture to study the response of cell behaviors to biochemical, topological and mechanical cues was becoming a new hotspot in this field, which coincided with the fact that stem cells research was a hotspot in biomedical field (Akhmanova et al., 2015). Another emerging research hotspot was mucosal delivery, which is related to nanotechnology, and TPP happens to be the most commonly used 3DP technology at the micro- and nanoscales (Laird et al., 2021). The map of keywords trend topics shows that microneedles (MN) have recently emerged as a new focus. An article demonstrated the use of TPP to produce MN array templates from which the silicone MN array molds were fabricated, finally, the molds were used to produce dissolving and hydrogel-forming MN arrays (Cordeiro et al., 2020). This shows that TPP is not only used to fabricate “direct” biomedical devices such as tissue engineering scaffolds but also can be used to manufacture “indirect” devices to produce biomedical structures in the biomedical field. It happened that there was a similar case, another article shown by the recent citation burst reference described ultracompact compound lens systems fabricated by TPP (Engelhardt et al., 2011). These unprecedented high-quality lens systems are also not “direct” biomedical devices, but they can have a tremendous impact on biotechnology and medical engineering in endoscopy, high-performance imaging and other aspects. As a subsequence, we can predict that using TPP as a sourcing technique to fabricate biomedical related structures and devices may be a new direction for the applications of TPP in the biomedical field. Finally, the timeline of the co-cited references shows a research frontier, magnetic actuation, and this was just right the content of a recent paper, which used TPP to make magnetic driven microswimmers for biomedical nanorobots (Wang et al., 2018). At the same time, it tells us that the research of functional polymers was a frontier topic of TPP biomedical applications.

However, our study has certain limitations. Firstly, the targeted articles focused on the applications of TPP in the biomedical field, and the searching and screening strategy could be improved to screen and include more studies on the applications of TPP. Secondly, machine algorithms on which most of the analysis in this article inevitably led to the problem of insufficient human intervention. For example, multiple synonyms led to the duplicates being included because the overlap between different clusters of keywords, and the author’s homonyms. Lastly, some of the most recent publications may not be extracted due to the search ending on 1 August 2022.

## Conclusion

This bibliometric analysis offered a comprehensive understanding of publications regarding the applications of TPP in biomedical field from 2003 to 2022, which could supply the references to us. The research concerning the applications of TPP in the biomedical field has a very promising research prospect as the number of relevant publications is growing constantly. As an interdisciplinary research scope, the development of this area calls for the immense cooperation among researchers and experts excelling in physics, materials science, chemistry, biomedical science, and mechanical engineering. Making better utilization of TPP as an additive manufacturing technology to fabricate high-resolution, well-defined and complex 3D structures has always been the research focus in this field. With stem cells behaviors and mucosal delivery becoming the focuses of biomedical research, TPP manufacturing microstructures for the above research were becoming the hotspots of researchers. Using TPP as a source technique to fabricate biomedical related structures and devices was a new research direction. In addition, the research of functional polymers, such as magnetic driven polymers, was the frontier topic of TPP biomedical applications.

## Data Availability

The original contributions presented in the study are included in the article/supplementary material, further inquiries can be directed to the corresponding authors.
